# Complex Antiplatelet Therapy Management in Patients Requiring Urgent Interventions for Pseudoachalasia and Esophageal Obstruction

**DOI:** 10.7759/cureus.57209

**Published:** 2024-03-29

**Authors:** Charles Vallejo, Kushinga Bvute, Oscar L Hernandez

**Affiliations:** 1 Internal Medicine, Florida Atlantic University, Boca Raton, USA

**Keywords:** antiplatelet therapy, primary percutaneous coronary intervention (pci), plavix, gastroenterology and endoscopy, cangrelor

## Abstract

This case report describes the use of intravenous (IV) cangrelor as a transient tool for antiplatelet bridging therapy in a 70-year-old male with coronary artery disease and esophageal strictures who underwent recent percutaneous coronary intervention (PCI) and subsequently required a pre-oral endoscopic myomectomy (POEM) procedure. The patient was switched from oral clopidogrel to IV cangrelor drip prior to the procedure, which was successful in preventing stent thrombosis. The case highlights the potential benefits of IV antiplatelet therapy in patients unable to tolerate oral medications in the setting of esophageal obstructions following recent coronary stent placement in a critical care setting.

## Introduction

Of the three million individuals worldwide who undergo percutaneous coronary intervention (PCI) each year, approximately 7-17% require a noncardiac surgery (NCS) within a year of stent placement [1]. Many patients undergo gastrointestinal surgery and may require antiplatelet interruption, potentially increasing the risk of stent thrombosis, perioperative myocardial infarction, or both. Interventions on the esophagus pose an additional challenge as post-operative intolerance to oral intake requires the transition to intravenous (IV) medication. Cangrelor, a P2Y12 inhibitor approved by the FDA in 2015, poses as an alternative antiplatelet agent in the setting of nondeferrable surgery requiring oral antiplatelet discontinuation [2]. This case illustrates the complexity of preventing stent thrombosis in a patient with a recent PCI requiring an urgent intervention to bypass his esophageal obstruction.

## Case presentation

**Figure 1:**
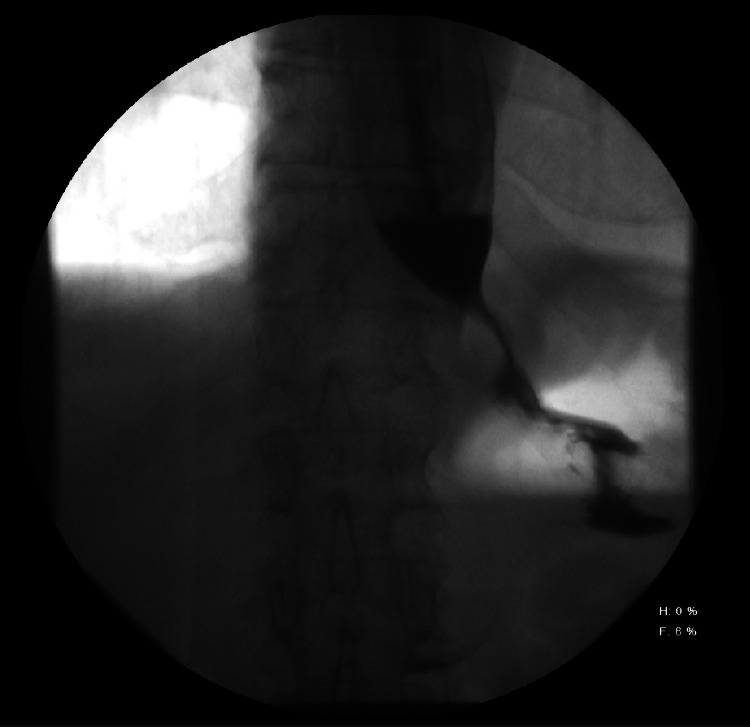
Esophagogram showing narrowing at the gastroesophageal (GE) junction

A 70-year-old male with a past medical history of chronic atrial fibrillation, coronary artery disease, and esophageal strictures presented with progressive dysphagia over the past six months along with a 65-pound weight loss. One month prior to admission he had two drug-eluting stents placed in his coronary arteries with oral antiplatelet therapy started with oral clopidogrel. Esophagogram on admission revealed narrowing at the gastroesophageal (GE) junction with a “bird beak” appearance suggestive of achalasia, as shown in Figure 1. Interventional gastroenterology was consulted, recommending a pre-oral endoscopic myomectomy (POEM) procedure. Oral clopidogrel was stopped five days before the procedure, with IV cangrelor bridging therapy begun six days prior to the POEM procedure while under observation in the ICU.

IV cangrelor drip was held one hour prior to the POEM procedure. Upper endoscopy revealed a GE junction infiltrating neoplasm with high-grade GE junction stenosis, resulting in pseudoachalasia. The tumor extended into the lesser curvature of the stomach with an infiltrating pattern, endoscopic ultrasound stage uT3N1Mx. An esophagogastric stent was placed for palliation. IV cangrelor drip was restarted two hours post-procedure. Due to severe nausea with the patient unable to tolerate oral medication post-procedure, he remained in the ICU on the IV cangrelor drip for three days post-op. He was able to resume oral clopidogrel on day three post-op. The patient remained stable and was later transferred to the floor for further recovery.

## Discussion

The risk of stent thrombosis is inversely proportional to the timing of NCS after PCI. Fortunately, IV cangrelor has a rapid onset, short plasma half-life, and more reliable antiplatelet action for acute interventions, such as in our patient whose oral intake was limited to 20 ounces of fluid daily [3]. In NCSs requiring patients not to take anything by mouth, it can be challenging to continue anticoagulation in the setting of recent coronary stent placement [4].

Given the success seen in this patient being transitioned from oral antiplatelet therapy to IV antiplatelet therapy prior to surgery, it can be assumed that IV antiplatelet therapy provides a therapeutic benefit in patients undergoing NCS in the setting of recent stent placement. This patient was of particular interest given his inability to tolerate oral medication in the setting of obstructive achalasia due to an esophageal mass.

## Conclusions

When comparing oral versus IV antiplatelet therapy in the setting of recent coronary stent placement for NCS, intravenous antiplatelet therapy can serve as a transient tool in those patients unable to tolerate oral medications in the setting of esophageal obstructions. This case illustrated a possible bridging strategy through the successful use of IV cangrelor in the ICU setting. This case may serve as an example of the many therapies available for clinicians when faced with antiplatelet bridging strategies on a recent PCI patient requiring esophageal interventions unable to tolerate oral intake.
